# Bidirectional associations between activity-related parenting practices, and child physical activity, sedentary screen-based behavior and body mass index: a longitudinal analysis

**DOI:** 10.1186/s12966-017-0544-5

**Published:** 2017-07-06

**Authors:** Ester F. C. Sleddens, Jessica S. Gubbels, Stef P. J. Kremers, Eline van der Plas, Carel Thijs

**Affiliations:** 1 Department of Health Promotion, NUTRIM School of Nutrition and Translational Research in Metabolism, Maastricht University, Maastricht University Medical Center, P.O. Box 616, 6200 MD Maastricht, the Netherlands; 2 Department of Epidemiology, Maastricht University, CAPHRI Care and Public Health Research Institute, P.O. Box 616, 6200 MD Maastricht, the Netherlands

**Keywords:** Activity-related parenting practices, Bidirectional associations, Body mass index, Children, Physical activity, Sedentary screen-based behavior

## Abstract

**Background:**

It has been generally assumed that activity-related parenting practices influence children’s activity behavior and weight status. However, vice versa parents may also change their parenting behaviors in response to their perceptions of their child’s activity behavior and weight status. This study examined the bidirectional relationships between activity-related parenting practices, and physical activity, sedentary screen-based behavior, and body mass index (BMI) between children’s age of 5 and 7 years.

**Methods:**

Three scales of the Activity-related Parenting Questionnaire (i.e. ‘restriction of sedentary behavior’, ‘stimulation of physical activity’, and ‘monitoring of physical activity’) were completed by 1694 parents of the Dutch KOALA Birth Cohort Study at the child’s age of around 5 and again around age 7. Physical activity, sedentary screen-based behavior and BMI were measured at both ages as well. Linear regression models were used to estimate the bidirectional associations between each parenting practice and the child’s physical activity levels, sedentary screen-based behavior and BMI z-scores.

**Results:**

Several parenting practices at age 5 predicted child physical activity, sedentary screen-based behavior, and BMI z-scores at age 7. Restriction of sedentary behavior positively predicted child BMI and sedentary screen-based behavior, whereas this practice negatively predicted child physical activity. In addition, stimulation of physical activity at age 5 was significantly associated with higher levels of child physical activity at age 7. The following child factors at age 5 predicted parenting practices at age 7: Child physical activity positively predicted parental stimulation of physical activity and monitoring activities. Sedentary screen-based behavior was associated with lower parental stimulation to be active.

**Conclusions:**

Findings generally revealed that parents and children mutually influence each other’s behavior. A reinforcing feedback loop was present between parental stimulation of physical activity and child physical activity. Bidirectional parent-child interaction should be considered in future research in order to properly inform parenting-related intervention programs aimed at preventing or treating childhood overweight or obesity. System dynamic methods to explore the existence of reinforcing or balancing loops are needed in this regard.

## Background

Parenting practices are content-specific acts of parenting [1], such as rules about activity behaviors. The literature on parenting and childhood obesity is dominated by studies about specific food-related parenting practices [2], whereas physical activity-related parenting practices are less studied but gaining interest during the last few years. Xu, Wen & Rissel [3] conducted a review about associations of parental influences with children’s physical activity and screen time, and found that parental encouragement and support increase children’s physical activity. In addition, reducing parents’ own screen time can lead to decreased child screen time. Earlier, two reviews were conducted on the then available activity-related parenting practices measures and associations with either physical activity or screen time [4, 5]. They found mixed evidence regarding the association between activity-related parenting practices and child physical activity and screen time [4, 5]. Despite limitations in existing studies, findings from a previous systematic review concluded based on their systematic review that supporting physical activity behaviors (including modeling) were positively related to children’s physical activity levels [5]. However, previous research hypotheses have been largely unidirectional, assuming that parenting practices induce child behaviors. In addition, most of these studies were cross-sectional in nature and therefore causality could not be assessed [5].

‘The chicken or the egg’ causality issue has hardly been addressed in the study of parent-child interactions in the development of childhood obesity. A few researchers tried to disentangle this issue in the parental feeding domain (e.g., [6–9]) (although solely with child BMI as a predictor and outcome and not dietary behavior), but studies that address microsystem dynamics with regard to activity-related parenting are lacking to date. The studies of parent-child interaction in the feeding domain found that food parenting practices were often influenced by the child’s weight status, in addition to the food parenting practice influencing the child’s weight gain [7–9]. Despite these findings, the energy balance-related parenting literature is dominated by studies characterizing parents as the agents acting upon the child, without considering children’s influence on parents [10, 11].

The aim of this study was to examine bidirectional associations between activity-related parenting practices (i.e., restriction of sedentary behavior, stimulation of physical activity, and monitoring of child physical activity), child physical activity and sedentary screen-based behavior, and parental perception of child weight status at age 5 and age 7. Based on previous food-parenting studies, we hypothesized parenting practices to be a response to child activity behavior and Body Mass Index (BMI), in addition to being a cause. The longitudinal design made it possible to examine the bidirectional nature of the parent-child dynamics: whether activity-related parenting practices predicted change in child activity behavior and BMI; or whether child activity behavior and BMI predicted change in activity-related parenting practices.

## Methods

### Data collection and participants

Data were collected within the KOALA Birth Cohort Study in the Netherlands (described elsewhere) [12]. Briefly, from 2000 onwards, healthy pregnant women from the general population who participated in an existing cohort study on pregnancy-related pelvic girdle pain were recruited (*N* = 2343; referred to as the ‘conventional’ recruitment group). In addition, healthy pregnant women with an ‘alternative’ lifestyle with regard to dietary habits (e.g., preferring organic foods), vaccination programs and/or antibiotics use (*N* = 491; referred to as the ‘alternative’ recruitment group) were recruited through several ‘alternative’ circles like anthroposophical physician offices and midwives, and organic food shops. All participants (*N* = 2834) were enrolled between 14 and 18 weeks of gestation and completed questionnaires during pregnancy and at regular intervals after birth. Informed consent was signed by all parents, and ethical approval was obtained from the Maastricht University/University Hospital Maastricht medical ethics committee.

For the current study we used data from the measurements around the child’s age of 5 and again around age 7. In total, 2061 participants (72.7% of the initial cohort) completed the questionnaire around age 5 and 1810 participants (63.9% of the initial cohort) completed the questionnaire around the child’s age of 7. Of those, 1694 completed the child’s parent-reported physical activity behavior questions and/or the activity-related parenting practices at both measurements. Children that were included in the final analyses had a somewhat higher BMI z-score at age 7 compared to children not included (−0.32 vs. -0.53, *p*-value = 0.03). There was no other selective drop-out (all *p*-values >0.05) with regard to recruitment group or other child or parental background characteristics. Child mean (*SD*) age during the first measurement around age 5 was 5.00 (0.53) years (range 3 to 6 years), and during the second measurement around age 7 was 7.17 (0.65) years (range 5 to 8 years).

### Measures

At both measurements similar questions were asked regarding activity-related parenting practices, child physical activity behavior and child weight and height.

#### Activity-related parenting practices

We used the Activity-related Parenting Questionnaire [13], which was based on the Child Feeding Questionnaire [14] and adapted to assess physical activity and sedentary screen-based behavior parenting practices. Three scales from this questionnaire were used for the current study: ‘restriction of sedentary behavior’ (assessing the extent to which parents restrict their child’s access to sedentary activities; 7 items), ‘stimulation to be physically active (assessing the extent to which parents encourage their child to be physically active; 3 items)’ and ‘monitoring activity (assessing the extent to which parents oversee their child's physical activity; 2 items)’. In the current study we decided to delete the item ‘As a reward for good behavior, I put on a nice video/DVD/computer game for my child’ originally belonging to the scale labeled ‘restriction of sedentary behavior’. This item was dropped prior to the analyses based on potential mismatch with this scale. For all three scales, a five-point Likert scale has been used ranging from 1 (*Completely disagree*) to 5 (*Completely agree*) for ‘restriction of sedentary behavior’ and ‘stimulation to be active’, and ranging from 1 (*Never*) to 5 (*Always*) for ‘monitoring activity’. The items used to assess these activity-related parenting practices and the corresponding Cronbach’s alphas at both measurements are listed in Table 1. Intercorrelations between the different parenting practices were low to moderate, ranging from 0.19 to 0.33.

**Table 1:** Descriptive and scale information of the activity-related parenting practice scales (*N* = 1694)

Activity-related parenting practices	Cronbach’s alpha; Mean (SD)	
	Age 5 years	Age 7 years
Restriction of sedentary behavior - I have to be sure that my child does not… * …watch too much television. * …play too many computer games. - If I did not guide or regulate my child’s activity behavior, (s)he would.. * watch too much television or play too many computer games. * not get enough physical activity. * be sedentary a lot (or play without being physically active). - I intentionally keep my child away from the television or computer.	0.71; 3.00 (0.76)	0.72; 3.12 (0.75)
Stimulation to be active - If my child says “I don’t feel like walking or bicycling to there”, I try to get him/her to do this anyway. - I have to be careful that my child gets enough exercise. - I make sure that my child travels actively on foot or by bicycle (with or without me) as often as possible.	0.58; 4.26 (0.65)	0.56; 4.45 (0.53)
Monitoring activity How much do you keep track of… - ..the amount of television your child watches and how many computer games (s)he plays? - ..the amount of physical activity your child has?	0.64; 3.94 (0.79)	0.57; 4.00 (0.67)

The Corrected-Item Total Correlations for each of the items were above 0.30; The item ‘as a reward for good behavior, I put on a nice video/DVD/computer game for my child’ originally belonging to the scale labeled ‘restriction of sedentary behavior’ was already dropped prior to the analyses based on theory; response scale ranging from 1 (*Completely disagree*) to 5 (*Completely agree*) for ‘restriction of sedentary behavior’ and ‘stimulation to be active’, response scale ranging from 1 (*Never*) to 5 (*Always*) for ‘monitoring activity

#### Child activity behavior and body mass index

Children’s activity behavior was assessed using questions based on a standard questionnaire for measuring activity behavior, used in Dutch Youth Health Care [15]. Parents were asked on how many days in a normal week during the last 4 weeks their child went to school by bicycle or on foot, had played sports at a sports club, and had played outside (outside school hours). A second question assessed the average duration of each of these activities, with five predefined answering categories ranging from half an hour or less, to 3 h or more. Based on the assumption that there is a linear association between physical activity and parenting practices [5], we recoded the answer scale to calculate the average time of these activities. Half an hour or less was recoded into 15 min, half an hour to 1 h into 45 min, 1 to 2 h into 90 min, 2 to 3 h into 150 min and finally, 3 h or more into 181 min. For the item ‘child went to school by bicycle or on foot’ answering categories were similar as above for age 7, but different for the measurement at age 5. The answering categories at age 5 for this item were: shorter than 10 min (recoded into 5 min), 10–20 min (recoded into 15 min), 20–30 min (recoded into 25 min), half an hour-1 h (recoded into 45), and more than 1 h (recoded into 61 min). The duration and number of days were multiplied to calculate the number of minutes spent on a particular activity per week. The number of minutes spent on the various activities were then added up to calculate the total number of minutes of physical activity per week, which was divided by 7 to get the average time (in minutes) the children were physically active per day. Sedentary screen-based behavior was assessed in a similar manner, asking parents about their child’s television watching (including videos and DVDs) and computer playing (including game consoles). A similar approach was followed as for physical activity to calculate the number of screen hours per day.

During both measurements, parents were asked to report their child’s weight and height (measured without shoes and clothes, specified to one decimal), in order to calculate the child’s BMI (weight (kg)/(height (m)^2^). BMI was then recoded into BMI z-scores compared to the 1997 national reference population (i.e., the Fourth Dutch National Growth Study) [16]. BMI z-score > 85th percentile was considered to indicate overweight and a BMI z-score > 95th percentile was considered to indicate obesity [17]. Anthropometric scores were systematically checked for plausibility using the following procedure. All outliers (z-scores >3, i.e. more than 3 standard deviations from the mean) for height, weight and BMI were identified. In addition, longitudinal anthropometric data were checked for inconsistencies and impossible and highly unlikely changes (e.g. a child shrinking in height). The outliers and inconsistencies were then checked with the original paper questionnaires. If the outlier could not be corrected based on this check, it was recoded into a missing value.

#### Background variables

As child covariates we included birth weight (in grams) and gender. With regard to parental covariates we included employment (i.e., the number of working hours per week) of the questionnaire completer and partner, their country of birth, and the highest parental educational level attained in the household at the child’s age of 5. Educational level was recoded into three levels (low, medium and high), in line with international classification systems [18]. Country of birth was recoded into ‘Netherlands’ versus ‘other’. We additionally included the recruitment group (‘conventional’ versus ‘alternative’) as a covariate in all analyses.

### Data analyses

Statistical analyses were performed using the statistical software package IBM Statistics 21. Descriptive statistics were used for the background characteristics of the sample. Both internal reliability coefficients (Cronbach’s α) and Corrected Item–Total Correlations (CITC) were calculated for the scales of the Activity-related Parenting Questionnaire at both ages. A cut-off point of 0.50 for Cronbach’s α was used [19]. CITC values above 0.30 were regarded as ‘good’ [20].

To assess the bidirectional associations between activity-related parenting practices and the child’s physical activity, sedentary screen-based behavior and BMI, two sets of linear regression models were conducted. Each set of models examines one of the two assumed directions of the association. The first set of models (A) examined the association between each of the activity-related parenting practices at age 5 and child’s physical activity, sedentary screen-based behavior and BMI z-scores at age 7. The second set (B) examined the associations between the child’s physical activity, sedentary screen-based behavior, and BMI at age 5 and each of the parenting practice scales at age 7. For both directions, three models are presented: Model 1: an unadjusted model; Model 2: a model adjusted for covariates (recruitment group; child birth weight and gender; parental employment, country of birth and educational level); and Model 3: a model adjusted for covariates and additionally for either baseline physical activity, sedentary screen-based behavior, or BMI z-score (in the models examining parenting practices as a predictor of child behavior and BMI (A)), or for baseline parenting practices (in the models examining child behavior as a predictor for later parenting practices (B)). Controlling for these baseline scores at age 5 in this third model allows for examination of the effect of changes within the dependent variable between ages 5 and 7 years, independent of the child’s baseline scores on the dependent variable.

## Results

### Sample characteristics

The percentage boys and girls taking part in the study were about equally distributed (Table 2). Most families were included in the category with the highest education level achieved (67.8%). The majority of the questionnaires were completed by mothers (97.6%). The majority of participants were of Dutch origin (more than 96% for both female and male caregivers). The children’s mean (*SD*) physical activity in minutes per week was 610.81 (394.25) at age 5 and increased to 904.48 (375.42) at age 7. For sedentary screen-based behavior this was 363.16 (292.19) minutes per week at age 5 and increased to 553.08 (351.69) at age 7. Mean (*SD*) BMI z-score at age 5 was −0.26 (0.99), compared to −0.32 (0.91) at age 7. To specify, 8.9% of the children were identified as being overweight/obese at age 5, whereas this decreased to 6.4% at age 7.

**Table 2:** Sample Characteristics (*N* = 1694)

				Age 5 years			Age 7 years		
Description		*n*	%	*n*	%	Mean (SD)	*n*	%	Mean (SD)
*Child gender*	Boy	868	51.2						
	Girl	826	48.8						
*Questionnaire completer*	Mother			1652	97.5		1619	95.6	
	Father			38	2.2		40	2.4	
	Unknown/other/NA			4	0.3		35	2.1	
*Partner questionnaire completer*	Mother			33	1.9		39	2.3	
	Father			1492	88.1		1436	84.8	
	Unknown/other/NA			169	9.9		219	12.9	
*Working hours questionnaire completer*						21.25 (9.25)			
*Working hours partner questionnaire completer*						36.71 (12.84)			
*Education level (highest in household)*	Low Medium High			72 472 1145	4.3 27.9 67.8				
*Birth country female caregiver*	Netherlands			1634	96.9				
	Other			52	3.1				
*Birth country male caregiver*	Netherlands			1622	96.3				
	Other			62	3.7				
*Recruitment channel*	Conventional Alternative	1372 322	81.0 19.0						
*Total physical activity (minutes per week)*				1676		610.81 (394.25)	1623		904.48 (375.42)
*Active transport to school (minutes per week)*				1679		45.10 (53.11)	1659		108.99 (106.12)
*Sports (minutes per week)*				1694		42.94 (50.82)	1655		127.62 (110.23)
*Playing outside (minutes per week)*				1677		522.42 (385.30)	1661		669.08 (319.46)
*Total sedentary screen-based behavior (minutes per week)*				1679		363.16 (292.19)	1659		553.08 (351.69)
*Television watching (minutes per week)*				1679		315.71 (258.45)	1671		417.06 (263.44)
*Computer playing (minutes per week)*				1681		47.39 (91.23)	1665		137.26 (163.50)
*Body Mass Index z-scores*				1585		−0.26 (0.99)	1446		−0.32 (0.91)
*Weight categories*	Underweight			132	8.3		102	7.1	
	Normal-weight			1312	82.8		1251	86.5	
	Overweight			97	6.1		67	4.6	
	Obesity			44	2.8		26	1.8	

Missings educational level 5, missings BMI (5 years) 109, missings BMI (7 years) 248, missings physical activity (5 years) 18, missing physical activity (7 years) 71, missing sedentary screen-based behavior (5 years) 15, missings sedentary screen-based behavior (7 years) 35, missings birth country female caregiver 8, missings birth country male caregiver 10

### Associations between activity-related parenting practices at age 5 and child BMI/physical activity/sedentary screen-based behavior at age 7

**Fig. 1:**
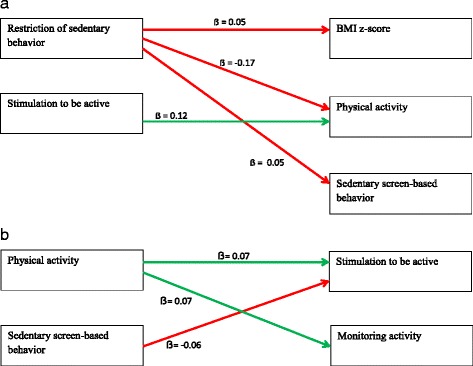
**a** A visual representation of the significant associations between activity-related parenting practices at age 5 and child’s BMI, physical activity and sedentary screen-based behavior development up to age 7. *Legend:* The direction of the standardized regression coefficient is presented in the figure, only for the third model (adjusted for covariates ‘recruitment group (conventional vs alternative), child gender and birth weight, parental educational level, employment and country of birth’ and in addition for BMI z-score at age 5 in parenting practice-BMI associations, and for either physical activity sedentary screen-based behavior in the parenting practice-physical activity/sedentary screen-based behavior associations; red line: unintended effects, green line: intended effects. **b** A visual representation of the significant associations between physical activity-related variables at age 5 and activity-related parenting practices development up to age 7. *Legend:* The direction of the standardized regression coefficient is presented in the figure, only for the third model (adjusted for covariates ‘recruitment group (conventional vs alternative), child gender and birth weight, parental educational level, employment and country of birth’ and in addition for restriction of sedentary screen-based behavior at age 5 in the activity-related variables & BMI-restriction of sedentary screen-based behavior association, and for either stimulation to be active or monitoring activity in the activity-related variables & BMI- stimulation to be active or monitoring activity parenting practice associations; Green line: reinforcing effect, red line: reactance effect

For a visual inspection of the significant associations found between activity-related parenting practices at age 5 and child BMI, physical activity and sedentary screen-based behavior development up to age 7 we refer to Fig. 1a. The parenting practice ‘restriction of sedentary behavior’ showed positive associations with BMI z-score and sedentary screen-based behavior at age 7 (Table 3), even after correction for potential confounders and for the child’s behavior at age 5 (i.e., for BMI z-score at age 5 when BMI at age 7 was predicted and for child sedentary screen-based behavior at age 5 when sedentary screen-based behavior at age 7 was predicted). In the final model (model 3), the association with sedentary screen-based behavior was marginally significant (*p* = 0.055). Restriction of sedentary behavior was negatively associated with the child’s physical activity level at age 7. Stimulation to be active was only related to the child’s physical activity. Higher scores on stimulation to be active were significantly associated with an increase of mean scores of the child’s physical activity 2 years later. In addition, parental monitoring of their child’s activity was not associated with children’s BMI, physical activity levels or sedentary screen-based behavior.

**Table 3:** Association between activity-related parenting practices at age 5 and child’s BMI, physical activity and sedentary screen-based behavior development up to age 7

		Standardized regression coefficients (β)		
Activity-related parenting practices	Model	BMI z-score	Physical activity	Sedentary screen-based behavior
Restriction of sedentary behavior	1	0.13***	−0.25***	0.07*
	2	0.13***	−0.23***	0.08**
	3	0.05*	−0.17***	0.05^a^
Stimulation to be active	1	−0.02	0.16***	0.00
	2	−0.02	0.15***	−0.03
	3	−0.02	0.12***	0.01
Monitoring activity	1	−0.04	−0.01	−0.05
	2	−0.04	−0.02	−0.03
	3	0.00	−0.03	0.00

* *p* < 0.05, ** *p* < 0.01, *** *p* < 0.001; a the *p*-value was marginally significant: *p* = 0.055; model 1: unadjusted; model 2: adjusted for recruitment group (conventional vs alternative), child gender and birth weight, parental educational level, employment and country of birth; model 3: adjusted for covariates from model 2 and in addition for BMI z-score at age 5 in parenting practice-BMI associations, and for either physical activity or sedentary screen-based behavior at age 5 in the parenting practice-physical activity/sedentary behavior associations; Explained variances (R2): for BMI: Model 1 = 0.01, Model 2 = 0.06, Model 3 = 0.39; for Physicalactivity: Model 1 = 0.08, Model 2 = 0.13, Model 3 = 0.21; for Sedentary screen-based behavior: Model 1 = 0.01, Model 2 = 0.13, Model 3 = 0.38

### Associations between child BMI/physical activity/sedentary screen-based behavior at age 5 and activity-related parenting practices at age 7

For a visual inspection of the significant associations found between physical activity-related variables at age 5 and activity-related parenting practices development up to age 7 we refer to Fig. 1b. For child BMI z-score (Table 4), significant associations were only found with the parenting practice ‘restriction of sedentary behavior’: this association was positive in the unadjusted model (model 1) and the model adjusted for covariates (model 2). The child’s physical activity was significantly related to all three activity-related parenting practices: positive for ‘stimulation to be active’ and ‘monitoring activity’, and negative for ‘restriction of sedentary behavior’. The association between physical activity and restriction of sedentary behavior at age 7 attenuated after adjustment for restriction of sedentary behavior at age 5, indicating that physical activity did not influence change of this parenting practice between age 5 and 7. The child’s sedentary screen-based behavior only had a significant association with stimulation to be active. Parents of children that were sedentary at age 5 were less likely to stimulate their child to be physically active at age 7. This association remained significant after correcting for potential confounders and considering the parenting practice ‘stimulation to be active’ at age 5.

**Table 4:** Association between physical activity-related variables at age 5 and activity-related parenting practices development up to age 7

		Standardized regression coefficients (β)		
Physical activity related variables	Model	Restriction of sedentary behavior	Stimulation to be active	Monitoring activity
BMI z-score	1	0.08**	0.04	0.02
	2	0.08**	0.04	0.02
	3	0.03	0.03	0.02
Physical activity	1	−0.11***	0.11***	0.07*
	2	−0.12***	0.11***	0.07*
	3	−0.03	0.07**	0.07*
Sedentary screen-based behavior	1	0.00	−0.07**	−0.05
	2	0.02	−0.09**	−0.04
	3	0.00	−0.06*	−0.03

* *p* < 0.05, ** *p* < 0.01, *** *p* < 0.001; model 1: unadjusted; model 2: adjusted for recruitment group (conventional vs alternative), child gender and birth weight, parental educational level, employment and country of birth; model 3: adjusted for covariates from model 2 and in addition for restriction of sedentary behavior at age 5 in the activity-related variables & BMI-restriction of sedentary behavior association, and for either stimulation to be active or monitoring activity in the activity-related variables & BMI-stimulation to be active or monitoring activity parenting practice associations. Explained variances (R^2^): for Restriction of sedentary behavior: Model 1 = 0.02, Model 2 = 0.06, Model 3 = 0.31; for Stimulation to be active: Model 1 = 0.02, Model 2 = 0.04, Model 3 = 0.20; for Monitoring activity: Model 1 = 0.01, Model 2 = 0.01, Model 3 = 0.16

## Discussion

This study examined the bidirectional associations between activity-related parenting practices (i.e., restriction of sedentary behavior, stimulation to be active, and monitoring activity) and the child’s BMI, physical activity levels, and sedentary screen-based behavior. To date, the focus in the literature has very much been on activity-related parenting practices as a risk factor for low levels of physical activity and high levels of sedentariness among children or high body weight. Our study reveals that children influence parenting as much as the other way around behavior.

In this study we found that child sedentary screen-based behavior negatively influenced stimulation of physical activity. In cross-sectional studies such a finding could easily be misinterpreted as parental stimulation to be active having a detrimental impact on children’s sedentary screen-based behavior, for example when they push children too much. However, our results suggest the reverse is true. It thus seems that parents of children with high levels of sedentary screen-based behavior do not stimulate their children (anymore) to be physically active. Parents might reach a point where unsuccessful efforts te decrease their child’s sedentary time in the past, have led to decreased self-efficacy to address the child’s behavior, which in turn influences their parenting practices [21]. Alternatively, parents might not consider sedentarity as a problem, or perceive it as an unchangeable trait of the child.

Parents are additionally responding to child physical activity levels; parents were more likely to monitor their child’s activity when the child was already physically active. These parents are probably enthusiastic about their child’s willingness to be physically active and therefore supporting and motivating their child, which could be characterized by increased monitoring of their child’s activity behavior.

In addition, we found that stimulation of physical activity led to higher levels of physical activity, but also that physical activity was positively related to stimulation of physical activity. This process can be characterized as a reinforcing or positive feedback loop. A feedback loop is a path along which information can be traced from one point in a system, and back to the point of origin. Feedback loops are an essential feature of system dynamics modeling [22]. In the current study, we found strong indications for the existence of such positive feedback loops. This is notable, since traditional health behavior theories often assume homeostasis or negative feedback loops: low levels of child PA would lead to parental stimulation, which would lead to more PA. The existence of positive feedback loops like those uncovered in the current study, may particularly explain non-linearity of environment – behavior relationships in the area of childhood obesity [23]. It may accurately describe the process where parents and children unintentionally become caught into a negative spiral from which it is hard to escape (i.e. more sedentary behavior leading to less stimulation to be active, in turn leading to even more sedentary behavior, and so on). However, the findings with regard to stimulation of physical activity and actual physical need to be interpreted with caution, as both concepts have a slightly different focus as regards the type of physical activity (i.e. focus on active travel versus on physical activity in general, respectively).

Restriction of sedentary behavior was found to have a detrimental impact on child activity-related and BMI outcomes; it was associated with an increase of child BMI and a decrease in child physical activity behavior. In accordance with the literature about restriction in the feeding domain, where food restriction could lead to an increase of children’s desire and intake of forbidden foods [24], it could be that children who are restricted in the amount of screen time increase their preferences for these behaviors and act upon that by being less physically active. However, we did not find that parents adapted their restrictive parenting practices to deviations in child outcomes (i.e. BMI z-score, physical activity, and sedentary screen-based behavior). It thus seems that parents do not (further) restrict their child’s sedentary behavior when their child is overweight or less active.

### Study strengths and limitations

This study is unique in its study design. Data were prospectively collected in a large cohort, and activity-related parenting practices and child physical activity-related variables were measured similarly on two time points. Although the questionnaires that were used were previously validated [13, 15], they have been used in only a few studies. We recommend replication studies using other parenting questionnaires, investigating the bidirectionality of the association between child outcomes and other parenting practices (such as parental modeling of activities). Also as regards behavior other questionnaires, including measures of other behaviours, might be worthwhile, as our study was limited to screen-based sedentary activities. Other sedentary behaviors such as reading are an understudied topic. Parental efforts decrease sedentary time might merely cause shifts from one type of sedentary behavior to another. Our study is further limited as it relies on self-reports. This systematic bias may produce spurious results. For instance, parents are likely to underestimate their children’s weight and overestimate height, especially if their child was overweight or obese, whereas parents of underweight children tended to overestimate weight [25]. This could be a reason why our study reported lower percentages of overweight/obesity (8.9% at age 5 and 6.4% at age 7) compared to the Dutch reference population of children aged 5–7 (2009: ranging from 12.8% and 18.8% for overweight and from 2.0% to 3.4% for obesity) [26]. It is likely that the present study yielded underestimates of associations between the activity-related parenting practice scales and child physical activity-related and BMI variables and vice versa, because of the parental reported nature of this study. This dilution of effects could also be due to imprecision of measurements and possible misspecification of models through assumptions of linearity. Future efforts should address more measurement points. While mutual influence between parent and child is a repeated, continuous process of interactions and adaptations, this process may likely be non-linear. Future studies could also establish whether the time lag of activity-related parenting practices that influence child physical activity-related variables is similar or different from the time lag for child physical activity-related variables to influence activity-related parenting practices.

## Conclusions

This study addresses the importance of conducting longitudinal research to get insight into the bidirectional nature of parent-child relations with regard to activity-related behavior. Parents and children are continuously adapting to each other and future efforts should make further progress in this area by addressing system dynamics in the home setting. The mutual influence between parents and children in influencing their activity-related behaviors could lead parents to unintentionally and unconsciously become caught into a negative spiral from which it is hard to escape (i.e., reinforcing causal loop chains). It is important to give those parents support as regards raising awareness for such processes and parenting practices that they could apply to get their child to become more physically active.

## Abbreviations

BMI: Body Mass Index
CITC: Corrected Item–Total Correlations
PA: Physical activity
